# Evolutionary Insights into the Relationship of Frogs, Salamanders, and Caecilians and Their Adaptive Traits, with an Emphasis on Salamander Regeneration and Longevity

**DOI:** 10.3390/ani13223449

**Published:** 2023-11-08

**Authors:** Bin Lu

**Affiliations:** Chengdu Institute of Biology, Chinese Academy of Sciences, Chengdu 610041, China; lvbin@cib.ac.cn

**Keywords:** extant amphibians, evolution, regeneration, aging

## Abstract

**Simple Summary:**

Amphibians have unique traits, such as regeneration and longevity in salamanders, frequent vocalization in frogs, and degenerative vision in caecilians. The genetic basis of these traits is not well understood. This study aimed to investigate the genetic changes underlying these unique traits, especially salamanders’ regeneration and longevity, by comparing the genes of amphibians to other vertebrates. I found that salamander genomes have undergone accelerated adaptive evolution, especially for development-related genes. Several salamanders’ genes are under positive selection and/or share mutations with other long-lived and regenerative vertebrates, suggesting that these genes are important for these unique traits. This study could help us to better understand the mechanisms of regeneration and aging, which could lead to the development of new ways to improve human health and well-being.

**Abstract:**

The extant amphibians have developed uncanny abilities to adapt to their environment. I compared the genes of amphibians to those of other vertebrates to investigate the genetic changes underlying their unique traits, especially salamanders’ regeneration and longevity. Using the well-supported Batrachia tree, I found that salamander genomes have undergone accelerated adaptive evolution, especially for development-related genes. The group-based comparison showed that several genes are under positive selection, rapid evolution, and unexpected parallel evolution with traits shared by distantly related species, such as the tail-regenerative lizard and the longer-lived naked mole rat. The genes, such as *EEF1E1*, *PAFAH1B1*, and *OGFR*, may be involved in salamander regeneration, as they are involved in the apoptotic process, blastema formation, and cell proliferation, respectively. The genes *PCNA* and *SIRT1* may be involved in extending lifespan, as they are involved in DNA repair and histone modification, respectively. Some genes, such as *PCNA* and *OGFR*, have dual roles in regeneration and aging, which suggests that these two processes are interconnected. My experiment validated the time course differential expression pattern of *SERPINI1* and *OGFR*, two genes that have evolved in parallel in salamanders and lizards during the regeneration process of salamander limbs. In addition, I found several candidate genes responsible for frogs’ frequent vocalization and caecilians’ degenerative vision. This study provides much-needed insights into the processes of regeneration and aging, and the discovery of the critical genes paves the way for further functional analysis, which could open up new avenues for exploiting the genetic potential of humans and improving human well-being.

## 1. Introduction

**Figure 1:**
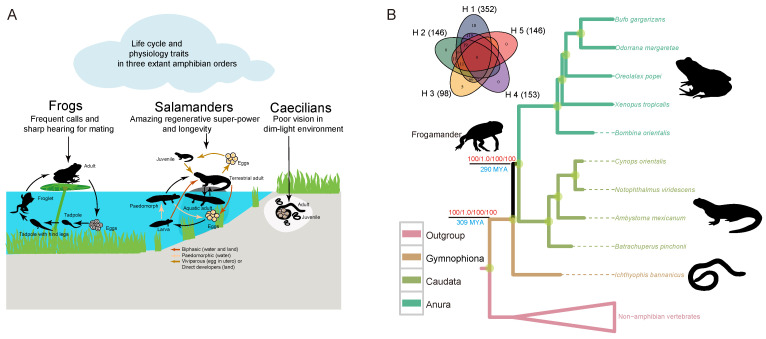
Physiology traits and phylogenomic analysis of extant amphibians. (**A**) The most prominent characteristics of frogs, salamanders, and caecilians. (**B**) Phylogenomic tree. The red numbers on the important nodes represent support values from the maximum likelihood (ML), Bayesian (PB), maximum pseudo-likelihood estimates (MPE), and maximum parsimony “Genes as characters” (GAC) methods. The blue numbers below the branch represent the origin date of the extant amphibians (309 MYA) and the living frogs and salamanders (290 MYA). Upper left is a Venn diagram of gene clusters supporting the different phylogenetic hypotheses, as shown in Figure S1. The numbers in brackets represent the quantity of genes that cannot reject a specific hypothesis.

Modern amphibians (Lissamphibia) possess some of the most intriguing features among vertebrates. Salamanders, including both aquatic larval axolotl and metamorphosed newts (*Cynops*; *Notopthalmus*; and *Pleurodeles*, the Iberian newt), have the remarkable ability to regenerate entire limbs, a tail, and even parts of the brain, eye, and heart [1,2]. However, there are some key differences in the regeneration modes of newts and axolotls. Newts regenerate lens and muscle tissues through dedifferentiation, while axolotls regenerate muscle tissues through stem cell activation [3,4,5,6]. It remains to be determined whether these differences reflect a higher degree of cell plasticity in newts. In addition, newts can regenerate more body parts than axolotls. Axolotls can only regenerate the eye lens during the first two weeks after hatching [5], while newts can regenerate the eye lens throughout their lifespan, and their ability to regenerate the lens does not decline with age or the number of lens removal/regeneration cycles [6]. Research using salamanders as a model system has gained tremendous insights into the developmental and physiological process of regeneration [7,8,9]. We now know that the extracellular matrix (ECM) plays a critical role in directing cell growth and migration, and nerves and immune cells are essential for regeneration [8,10,11,12]. When macrophages were removed, salamanders lost their ability to regenerate and instead formed scar tissue [8]. Other vertebrates, including human, lose their regenerative potential with age, largely because of failure to maintain tissue homeostasis [13]. Furthermore, salamanders have one of the longest life spans for their body size [14], which naturally raises the possibility of genetic interactions underlying regeneration and aging [15,16]. Other traits, such as the diverse body plans of modern amphibians, the vocalization of frogs, and many traits associated with the fossorial lifestyle of caecilians, are just as amazing (Figure 1A). However, the genetic mechanisms behind these traits remain largely elusive. In addition, persisting controversies surrounding the origin of Lissamphibians and the relationships among the three main groups have hindered comparative analysis [17].

The recent boom in genomic work provides an excellent opportunity to explore the genetic architecture of major evolutionary changes. Amphibians have only one published salamander genome [18], which is largely limited by their extremely large genome sizes. Alternative approaches, such as transcriptome sequencing, are commonly used [19,20]. The enormous amount of available genomic resources enables large-scale and in-depth comparative analyses. Furthermore, several distantly related vertebrates share some phenotypic traits with amphibians; for instance, lizards can regenerate tails, and naked mole rats and Brandt’s bats also have long lifespans [21]. This provides opportunities to examine potentially shared genetic mechanisms of the same trait. Although it has been argued that parallel/convergent evolution at the molecular level is often rare because there could be several genomic routes leading to the same phenotype, shared genetic changes would provide strong evidence for adaptive evolution [22].

Using bioinformatics and a comparative analysis of genomic data, I explored the potential genetic mechanisms of major traits in modern amphibians, in particular, the regenerative healing and anti-senescence capabilities of salamanders. My specific objectives were (1) to address the long-debated question of how the three orders of modern amphibians (Anura, Gymnophiona, Caudata) are related to each other as well as to other vertebrate groups; and (2) based on the resulting tree, I explored the genetic architecture of several traits of the three groups of modern amphibians, with a focus on the regeneration capacity and longevity of salamanders, as well as the vocalization and hearing of frogs and the degenerative visual function of caecilians.

## 2. Materials and Methods

### 2.1. Sample Collection, Transcriptome Sequencing, and De Novo Assembly

Transcriptome sequences for five amphibian species were acquired, including Yunnan caecilians (*Ichthyophis bannanicus*; collected in Jinghong, Yunnan Province, China, in 2018), Baoxing tooth toads (*Oreolalax popei*; collected from Baoxing County, Sichuan Province, China, in 2018), oriental fire-bellied toads (*Bombina orientalis*; collected from Qingdao, Shandong Province, China, in 2018), stream salamanders (*Batrachuperus pinchonii*; collected from Baoxing County, Sichuan Province, China, in 2018), and Chinese fire-bellied newts (*Cynops orientalis*; collected from Wuxue, Hubei Province, China, in 2018). All specimens were identified using a morphological method [23]. For evolutionary analyses, I collected samples from two adult individuals of each species, one male and one female. Individuals were euthanized and dissected immediately after death. RNA was separately extracted from the brain, liver, heart, skeletal muscle, and gonad tissues of these individuals using the Trizol protocols (Invitrogen, Carlsbad, CA, USA) and mixed in approximately equal quantities. For the limb regeneration experiment, adult newts were anesthetized and placed in a sterile dish. The right forelimb zeugopod was amputated using sterile scissors. The newts were then returned to their home tanks and monitored for recovery. The proximal healing tissue was harvested at 0 h, 1 day, 5 days, 10 days, and 20 days post-amputation, and RNA was separately extracted using the Trizol protocols (Invitrogen, Carlsbad, CA, USA). Three newts were used for each time window of limb regeneration. The concentration and integrity of total RNA was examined using agarose gel electrophoresis, a NanoPhotometer spectrophotometer (IMPLEN, Westlake Village, CA, USA), and an Agilent Bioanalyzer 2100 system (Agilent Technologies, Santa Clara, CA, USA). cDNA libraries were constructed and subsequently sequenced on an Illumina HiSeq2000 platform, which was carried out by Novogene Inc. (Beijing, China). Approximately 4 G of raw data of paired-end reads was obtained for each transcriptome.

Quality filtration and de novo assembly were performed. The raw reads were first cleaned by filtering out the adapter sequences using Trimmomatic v0.36 [24]. Reads with more than 10% unknown base calls and low-quality reads (<Q20) were also discarded. Preliminary assemblies were produced using Trinity v2.9.0 [25] according to the published protocols [26]. To ensure the quality of the assembly, I also performed a multiple K-mer and multiple coverage cutoff values assembly using ABySS v2.0 [27,28]. Combinations of five K-mer lengths (21, 31, 41, 51, and 61) and six coverage cutoff values (2, 3, 6, 10, 15, and 20) were used, and 30 raw assemblies were produced. Sequence overlaps and redundancies were then eliminated to produce the final assembly using the programs CD-HIT-EST v4.8.1 [29] and CAP3 v10.2011 [30].

### 2.2. Phylogenetic Analyses and Date Estimation

#### 2.2.1. Construction of the Raw Phylogenomic Datasets

Genomic data or transcriptome data of five additional amphibian species were obtained from previous studies, including the green odorous frog (*Odorrana margaretae*) [31], Asiatic toads (*Bufo gargarizans*) [32], axolotl (*Ambystoma mexicanum*) [33], eastern newt (*Notophthalmus viridescens*) [34], and western clawed frog (*Xenopus tropicalis*) [35]. A total of ten species were used to represent the three extant orders. Humans and the green anole were used as outgroups.

Putative orthologous genes were identified using the program HaMSTR v1.6.0 (Hidden Markov Model-based Search for Orthologs using Reciprocity) [36] based on its core ortholog database. Amino acid alignments were generated using Clustal Omega v1.2.4 [37] with the default parameters. Codon alignments were generated based on the amino acid alignments. A set of randomly sampled genes was selected, and their alignments were manually checked to ensure the performance of the program. Two raw supermatrix datasets were constructed, one by concatenating the coding regions of the nucleotide sequences (CDS) and the other by concatenating their corresponding amino acid sequences.

#### 2.2.2. Data Filtration

To remove or reduce potentially detrimental effects of several confounding factors in phylogenomic reconstruction, I filtered the data using four approaches. First, it is well known that uneven distributions of missing data can affect phylogenetic inferences [38,39]. I used the Alistat v1.7 to quantify the sparseness of the concatenated alignments. Second, the completeness score value ranging from 0 to 1 was estimated. Selecting informative subsets of supermatrices increases the chances of finding the correct trees [40]. I also used mare v0.1.2 [40] to assess the information content of genes in the supermatrices by measuring potential phylogenetic signals and data coverage. Genes with no information content were eliminated. Third, I investigated the influence of base compositional heterogeneity (CH) on the phylogenetic reconstruction using BaCoCa v1.1 [41]. The RCFV (Relative Composition Frequency Variability) values across all taxa were calculated for the complete datasets [42]. The higher the RCFV value, the higher the degree of compositional heterogeneity. Chi-square tests were conducted, and a significance level was set at 0.01, where a *p*-value below 0.01 indicates that the composition significantly deviates from homogeneity, according to the author’s suggestion. Heterogeneous genes were excluded from the supermatrices. Fourth, the common phylogenetic assumption assumes that evolutionary processes can be modeled and DNA sequences “evolved under globally stationary, reversible and homogeneous (SRH) conditions” [43,44,45]. In fact, CH across the sequences is common, and the evolutionary process is more complex than the model assumption. Non-SRH conditions will introduce systematic errors during the tree reconstruction process and even generate false phylogenetic conclusions [46,47]. I used the program SymTest to assess whether the concatenated sequences are consistent with evolution under SRH conditions. In addition, *p*-value heatmaps were generated to visualize which sequence pairs could be assumed to have evolved under SRH conditions and which ones violated this assumption.

After eliminating “noisy” genes and sites, select optimal data subsets were extracted from the raw supermatrices of both the CDS and amino acid sequence and then used in the phylogenomic analyses.

#### 2.2.3. Phylogenomic Tree Construction

I analyzed the two filtered supermatrices using both the maximum likelihood (ML) and Bayesian inference (BI) methods. ML trees were inferred using RAxML v8.0.19 [48] with the site-homogeneous JTT-F+G and GTR+G models, and the BI tree was inferred using PhyloBayes v3.3 [49] with the site-heterogeneous CAT-GTR model. The best-fit partitioning schemes and substitution models for the supermatrices were determined by PartitionFinder v2.1.1 [50]. The Bayesian Information Criterion (BIC) was chosen to compare partitioning schemes and models of molecular evolution.

I also applied ML approaches on each filtered gene to obtain all the individual gene trees. The tree sets were used to evaluate alternative phylogenetic hypotheses and to infer species trees.

I evaluated five phylogenetic hypotheses concerning the relationships among the three orders of modern amphibians. The first three hypotheses assume a monophyletic origin for Lissamphibians. (1) The Batrachia hypothesis proposes a frog–salamander sister relationship [51,52,53,54]. (2) The Procera hypothesis proposes a salamander–caecilian sister relationship [55,56,57]. (3) I also examined the frog–caecilian sister relationship, although no previous study has suggested the existence of this relationship. The last two hypotheses assume a paraphyletic origin for Lissamphibians, which were suggested by paleontological data. (4) Caecilians are more closely related to amniotes than to salamanders and frogs [58], while salamanders and frogs form a monophyletic group [59,60,61]. (5) Caecilians and salamanders are sisters, and together, they cluster with amniotes without frogs [62,63]. For each gene, RAxML was used to compute the per-site log-likelihood values of the five constrained topologies with the GTR+G substitution model. AU tests [64] were then performed with CONSE v 0.20 [65] to calculate *p*-values. A small *p*-value (0.05) for a topology indicates that the topology is significantly worse than the best one and should be rejected. For each hypothesis, I recorded the genes that could not be rejected by the AU tests. The *p*-values of each individual gene for each topology were converted into heatmaps using Phylcon v1.0 [66].

I also used two supertree approaches to construct species trees from individual gene trees. It is well known that gene trees are not always consistent with species trees due to incomplete lineage sorting and other biological reasons [47]. Here, I applied a pseudo-likelihood approach under the multi-species coalescent model [47,67] to overcome potential limitations and to obtain maximum pseudo-likelihood estimates (MPEs) of species trees from a collection of individual gene trees. I also used the parsimony-based “genes as characters” (GAC) approach [68] to infer species trees. This method treats each gene as a single-ordered multi-state character, and individual gene trees are described by step matrices. All the characters were weighted equally because no reasonable a priori information exists. I assumed that these genes evolved largely independently to satisfy the independent character assumption of parsimony analysis. Finally, parsimony analyses were performed in PAUP. A heuristic search was employed with TBR branch swapping and 100 random addition replicates. Bootstrap values were estimated with 1000 replicates.

#### 2.2.4. Divergence Time Estimates

Data were acquired for ten additional vertebrates, including zebrafish (*Danio rerio*), fugu (*Takifugu rubripes*), Amazon molly (*Poecilia formosa*), coelacanth (*Latimeria chalumnae*), Shedao pit-viper (*Gloydius shedaoensis*) [69], alligator (*Alligator mississippiensis*), chicken (*Gallus gallus*), opossum (*Monodelphis domestica*), elephant (*Loxodonta africana*), and mouse (*Mus musculus*). Most data were downloaded from Ensembl [70]. The dataset included 22 vertebrate species representing all major lineages. This dataset provided multiple calibration points, and it also formed the basis for all downstream analyses. 

Multiple calibration points provide more realistic divergence time estimates overall [71]; thus, I used four calibration points, including both soft minimum and maximum time constraints: (1) bird–mammal split (min 312.3 MYA, max 330.4 MYA); (2) human–toad split (min 330.4 MYA, max 350.1 MYA); (3) crocodile–lizard split (min 259.7 MYA, max 299.8 MYA); and (4) the origin of crown Osteichthyes (min 416.0 MYA, max 421.75 MYA). A relaxed molecular clock Bayesian method implemented in MCMCTREE v4.9 [72,73] in PAML v4.9 was used to estimate the divergence time among the three modern amphibian orders. I only used the 2nd codon of the CDS and amino acid datasets to estimate the divergence date to avoid potential problems associated with saturation. The results from molecular clock estimates were compared to the ages of Batrachian fossils from the early Permian.

### 2.3. Test for Selections on Lissamphibia

#### 2.3.1. Lineage-Specific dN/dS and dS Estimates

The evolutionary rate ratio of divergence at nonsynonymous and synonymous sites, dN/dS, is a widely used indicator to measure the magnitude of natural selection acting on protein-coding genes [74]. A lower dN/dS ratio indicates a strong purifying selection against protein changes, and an elevated dN/dS ratio suggests a weak purifying selection or a strong positive selection in favor of protein alterations. 

The dN/dS calculation was based on the 22-species dataset, which includes all major lineages of vertebrates and allows us to compare Lissamphibia to other vertebrates. I also constructed a reduced dataset with only the ten amphibians. This dataset has fewer taxa but more and longer orthologs, which allows us to perform an in-depth analysis of genes involved in adaptive and parallel evolution.

The dN/dS ratio for each lineage was estimated using a maximum likelihood approach [75], implemented in CODEML of the PAML 4 software [76]. The free-ratio branch model, which allows the dN/dS ratio to vary for different branches, was run on each ortholog and the concatenated supermatrices. Abnormal values (dN/dS > 5) were excluded from the analysis. A mean dN/dS value for each major group was calculated by averaging the ratio of all terminal branches within the group. I also calculated the number of synonymous substitutions (dS) to represent the rate of neutral evolution. Furthermore, I used PHAST v1.5 [77] to estimate the substitution rates for 4-fold degenerate sites in the concatenated supermatrices. 

#### 2.3.2. Rapidly Evolving GO Categories

The dN/dS ratio of the Gene Ontology (GO) category can partially reflect the evolutionary rate of a functional module. I identified rapidly evolving GO categories (REGOs) in the five major groups of vertebrates. During the transition from water to land, vertebrates experienced many major changes in their anatomic structure; therefore, I investigated the evolutionary pattern of developmental functions in extant amphibians and compared the proportion of REGOs involved in development between the major groups. I also compared development-related genes within each major group. Salamanders have an extended life span and remarkable regeneration ability during any stage of their life cycle; therefore, I further compared the adaptive evolutionary rate of GOs in modern salamanders with that in their ancestors. If development- and/or aging-related GOs accelerated adaptive evolution in living salamanders relative to their ancestors, this may suggest that salamanders’ superpower continually evolved and improved. Conversely, it may imply that these great changes occurred in the salamanders’ ancestors, and extant salamanders just inherited this ability. The Wilcoxon rank sum test was used to compare the dN/dS ratios of a particular gene or GO category to that of the other genes or categories as background.

#### 2.3.3. Identification of Genes under Positive Selection

The branch-site model implemented in the program CODEML v4.9 was used to detect positively selected genes (PSGs) along a specific lineage. The model assumes that foreground branches are under positive selection and background branches evolve in a neutral fashion. I compared the selection model (alternative model, dN/dS > 1) and the neutral model (null model, dN/dS = 1) using a likelihood ratio test (LRT). A chi-square test was conducted for each gene to assess statistical significance. Multiple testing was corrected by applying the false discovery rate (FDR) method. For a gene, if the selection model has a significantly higher likelihood than the neutral model (FDR-adjusted *p*-value < 0.05), this indicates that these genes on the foreground branch might have experienced positive selection.

#### 2.3.4. Identification of Fast-Evolving Genes

To identify the fast-evolving genes (FEGs) in Lissamphibia, I ran a one-ratio branch model and a multi-ratio branch model with CODEML in PAML to estimate the global and local dN/dS ratios, respectively. The one-ratio model assumes that all branches have been evolving at the same rate (null hypothesis), and the multi-ratio model allows the foreground branch to evolve at a different rate (alternative hypothesis). Salamanders, frogs, and caecilians were set as the foreground. The LRT was employed to compare the one-ratio and the multi-ratio branch models. The *p*-values of the chi-square test were adjusted using FDR correction for multiple testing. If a gene had an FDR-adjusted *p*-value of <0.05 and a higher dN/dS in the foreground branch than in the background branch, it was considered an FEG in the foreground branch. Functional enrichment analyses for FEGs were carried out using DAVID bioinformatics resources [78].

### 2.4. Test for Parallel Evolution

I tested patterns of parallel evolution between modern amphibians and three distantly related vertebrates, which share the characteristics of interest with amphibians and have relevant data. The green anole (*Anolis carolinensis*) is capable of regenerating its tail; the Brandt’s bat (*Myotis brandtii*) has longevity, super hearing for echolocation, and reduced visual capacity [79,80]; and the naked mole rat (*Heterocephalus glaber*) has longevity, cancer resistance, pain insensitivity, degenerated hearing, and poor visual perception [81,82,83,84,85]. In addition, zebrafish, chicken, and humans were also included for comparison. Ancestral sequence reconstruction was carried out using both ANCESTOR v1.0 [86] and CODEML. 

To reduce the influence of uncertainty and individuality, I only focused on amino acid changes shared by all members in a group (e.g., all salamanders examined). I first identified amino acid positions where changes only occurred in two lineages: an amphibian group and a distantly related vertebrate species that share the same phenotypic trait of interest. If they share the same amino acid residue and are derived from the same ancestral amino acid residue, these changes were defined as “parallel”. If the changes resulted in different amino acid states in the extant species, the changes were classified as “common”. Common changes may be a possible indicator of adaptation accomplished via multiple different amino acids at the same position (reference). CONVERG v2.0 [87] was used to compute the probabilities that the observed parallel substitutions are attributable to random chance. 

Genes with parallel changes or common changes were then compared to PSGs and FEGs, and overlapping genes were subjected to further analysis. Orthologs from other bats, including three echolocating bats—the little brown bat (*Myotis lucifugus*), David’s myotis (*Myotis davidii*), and vampire bat (*Desmodus rotundus*)—and one non-echolocating bat, the black flying fox (*Pteropus alecto*), were downloaded for comparison. The alignment quality of these candidate genes and the degrees of conservation of the region with amino acid changes were manually checked. Furthermore, candidate genes associated with human/mouse diseases were determined through exploring disease databases, such as OMIM (http://omim.org (accessed on 1 November 2022)) and GeneCards (www.genecards.org (accessed on 1 November 2022)) for human and MGI (http://www.informatics.jax.org (accessed on 1 November 2022)) for mouse, which focus on the relationship between disease phenotype and genotype. 

### 2.5. Prediction of Functional Impact of Variants

I used SIFT v6.2.1 [88], PROVEAN v1.1.5 [89], and PolyPhen-2 v2.2.2 [90] to predict the possible effect of unique amino acid substitutions and positively selected and parallel mutations on protein structure and function.

I retrieved information on protein domains and important sites and the 3D structure of proteins from the InterPro database [91] and RCSB PDB [92], respectively. Amino acid conservation scores across the sequences of candidate genes were calculated using the Rate4Site algorithm [93,94] on the ConSurf server [95]. The scores were converted by multiplying by −1 so that higher scores indicate higher conservation. Local average conservation was calculated by fitting a cubic smoothing spline to the per-site conservation scores using the smooth.spline method in R. Unique amino acid changes were mapped onto conservation domain plots and the protein 3D structure.

### 2.6. Quantitative Real-Time PCR

The proximal healing tissue was harvested at 0 h, 1 day, 5 days, 10 days, and 20 days post-amputation. RNA was extracted using the Trizol protocols (Invitrogen) and then reverse transcribed using a PrimeScript™ RT reagent Kit (Perfect Real Time, TaKaRa) according to the manufacturer’s instructions. The primers for OGFR were forward: 5′-CAGCCCAATGGTGTTCCTGAT-3′; and reverse: 5′-GCGGACAAACCTTTCTTTCA-3′. The primers for SERPINI1 were forward: 5′-ATTTAAGGGATCTATCTGAGGCC-3′; and reverse: 5′-CACCCAGCCATTGATGTGTT-3′. The primers for ACTIN were forward: 5′-AGATCTGGCACCACACCTTC-3′; and reverse: 5′-CAGTGGTACGACCAGAAGCA-3′. The qPCR reactions were performed on a BioRad CFX96 Real-Time PCR Detection System using the EvaGreen 2X qPCR MasterMix (Abm). The thermal cycling parameters were 10 min at 95 °C, followed by 40 cycles of 15 s at 95 °C, 30 s at 52 °C, and 30 s at 72 °C. Three replicates were performed at each time point, and failed reactions were excluded for the analysis. The relative expression level of unigenes was normalized to ACTIN as the comparative Ct and calculated using the delta delta Ct (2^−ΔΔ^Ct) method.

## 3. Results and Discussion

### 3.1. Robust Support for Frog–Salamander Sister Relationship

I gathered data from ten amphibian species to infer the origin of and relationships between three extant amphibian orders (Figure 1B). After filtering the one-to-one ortholog dataset, I ultimately utilized 369 coding sequences (CDSs) (second codon positions and first + second codon positions) and 772 protein sequences for the phylogenomic analyses. The datasets had relatively full coverage (completeness score: 0.7–1.0; Figure S2A), high information content (information content: 0.6–1.0; Figure S2B), and a low degree of CH (RCFV value < 0.025; Figure S3) and were least affected in terms of violation of the assumption of evolution under global SRH conditions (Figure S4).

Both the partitioned maximum likelihood (ML) and Bayesian inference (BI) analyses of the concatenated data strongly supported the monophyly of Lissamphibia and the frog–salamander sister relationship (the Batrachia hypothesis; Figure 1B). In addition, two independent supertree analyses, including the maximum pseudo-likelihood estimates (MP-EST) from a collection of individual gene trees [96] and the genes as characters approach (GAC) [68], both found the same topology as the concatenated data tree, providing strong support for the Batrachia hypothesis (Figure 1B). 

I also examined the conflicts among individual genes and detected the degrees of support of various genes for the alternative hypotheses. The AU test indicated that the Batrachia hypothesis (H1) was supported by the vast majority of the data, with 352 of 369 CDSs and 708 of 772 proteins supporting the hypothesis (Figure 1B and Figure S5), while the Procera hypothesis (H2), which posits that salamanders and caecilians are sister taxa, was supported by only 146 of 369 CDSs and 274 of 772 proteins. Surprisingly, the paraphyletic origin hypothesis (H4), which posits a close relationship between caecilians and amniotes and a sister relationship between salamanders and frogs, was supported by a large number of genes (153 of 369 CDSs and 374 of 772 proteins). The frog–caecilian sister relationship (H3) received the lowest support, with only 98 of 369 CDSs and 194 of 772 proteins supporting it.

Genome-scale data provide opportunities for resolving difficult phylogenetic relationships [97,98]. My data and analysis provide strong support for the monophyly of Lissamphibia and the salamander–frog sister relationship, consistent with the findings of recent studies [99,100,101].

### 3.2. Fossil-Compatible Divergence Time of Lissamphibia

**Figure 2:**
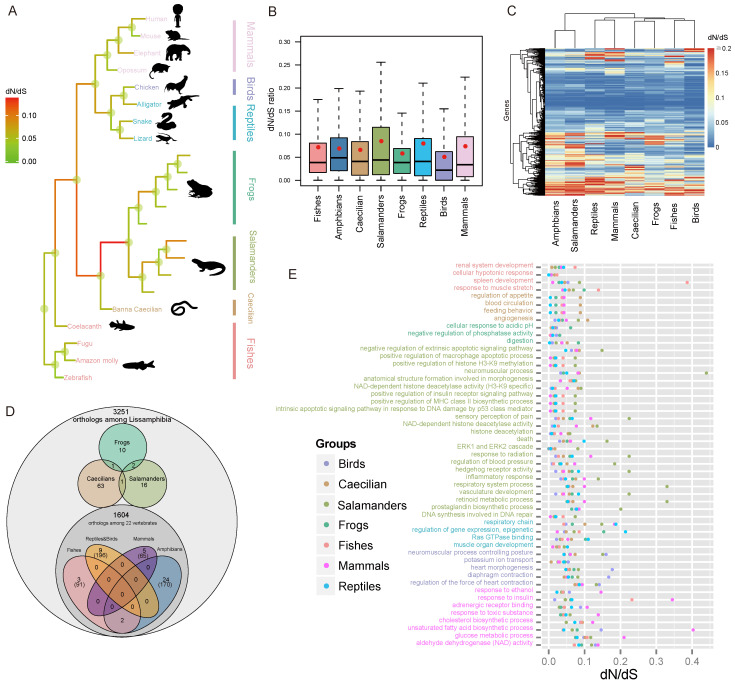
Accelerated rate of adaptive evolution in salamanders. (**A**) dN/dS ratio tree of 22 vertebrates shows that salamanders have a higher dN/dS ratio than any other vertebrate group. (**B**) The boxplot of dN/dS ratios for all genes in each major vertebrate group, which shows that salamanders have a much higher mean dN/dS ratio than any other group (*p* ≤ 0.025 for all pairwise tests). (**C**) The clustered heatmap of dN/dS ratios of major vertebrate groups also shows that salamanders are distantly related to other vertebrate groups in terms of dN/dS ratio. (**D**) Venn diagram showing number of PSGs and FEGs (in brackets) in Lissamphibia and other vertebrates. (**E**) The rapidly evolving GO categories (REGOs) in Lissamphibia and other vertebrates show that salamanders have a significantly higher number of REGOs than any other vertebrate group.

A total of 22 vertebrate species (Figure 2) were included in this analysis. I used four calibration points from vertebrates (Figure S6) to estimate the divergence time of Lissamphibia. The results indicate that modern amphibians arose in the late Carboniferous, about 309 MYA (Figure 1B and Figure S6). This is consistent with the fossil record; amphibian-like early temnospondyls (e.g., *Amphibiamus*) appeared in the Carboniferous [102]. The origin of Batrachia was estimated to take place in the early Permian (~290 MYA; Figure 1B and Figure S6). This is extremely close to the estimated age of the “frogamander”, *Gerobatrachus hottoni* (~290 MYA), from Texas during the early Permian [59]. *Gerobatrachus hottoni*, who possessed a large frog-like head and a salamander-like tail, was considered the closest relative of Batrachia [59]. Furthermore, the origins of living frogs and salamanders were dated back to the late Triassic (~200 MYA) and middle Jurassic (~170 MYA), respectively (Figure S6), which was close to or matched the fossil study well (~185 MYA for frog origin and ~170 MYA for salamander origin) [103]. 

My age estimates were younger than most previous molecular studies [59] but are mostly consistent with the fossil evidence. For example, the origin of Lissamphibia was estimated to be as young as the early Permian at 294 million years ago (MYA) [54] or as old as the late Devonian period at 369 MYA [104]. Most previous studies used only one or a few genes, which may contribute to the variation and discrepancy.

### 3.3. Rapid Evolution of Salamander Genes

To gain insight into the evolution of Lissamphibia, I compared aspects of their coding gene architecture to those of the other major vertebrate lineages (Figure 2A), including the evolutionary rate and genes or gene clusters that are potentially under positive selection. 

The dN/dS ratio has been widely used as a measurement of the rate of adaptive evolution. The colored dN/dS tree shows that salamanders exhibited a higher ratio of dN/dS relative to all the other vertebrate groups (Figure 2A). Furthermore, the distribution of dN/dS ratios among the salamander lineages was significantly larger than that of the other vertebrate groups (Wilcoxon rank sum test (WRST), *p* < 0.03 for all pairwise tests; Figure 2B). Additionally, a cluster analysis for the vertebrate dN/dS ratios found that salamanders were located on a lone branch at the base of the clustering tree and were very different from the other vertebrates (Figure 2C). Evidently, globally elevated evolutionary rates for protein-coding genes likely occurred in salamanders’ genomes. 

This accelerated evolution can either be due to selection (reduced purifying selection and/or increased positive selection in favor of protein alterations) or a high mutation rate, which can be measured as synonymous mutations [105]. 

To test whether salamanders have high mutation rates, I compared the mutation rates at synonymous sites (dS) among the major lineages of vertebrates, which has often been used to represent the neutral mutation rate. Interestingly, salamanders exhibited an extremely low dS compared to other vertebrates (Figure S7; WRST *p* < 2.9 × 10^−40^ for all pairwise tests). On average, the dS of salamanders was approximately one-third of the dS of caecilians and frogs. I further estimated rates for the four-fold degenerate sites (4D), which evolve the closest to the neutral rate [106]. As expected, the color pattern of the 4D mutation rate tree is very similar to that reconstructed using synonymous sites (Figure S8). 

Clearly, salamanders do not have elevated mutation rates, and the high dN/dS ratios are due to selection forces. I further explored aspects of selection forces below. Previous work showed that salamander mitochondrial genomes also display high dN/dS ratios, although their comparisons were restricted to salamanders and frogs [107]. 

I also noticed that both the common ancestors of Lissamphibia and Batrachia demonstrated higher dN/dS ratios than the extant amphibians (Figure 2A). These are likely a reflection of the rapid genomic changes required during their early adaptation to terrestrial life. 

### 3.4. Detection of Selection

I identified a series of fast-evolving genes (FEGs; Table S1) and positively selected genes (PSGs; Table S2) in salamanders, frogs, and caecilians (Figure 2D). The number of candidate genes was conservative because my analyses were conducted on groups of species rather than individual species. This approach helps to reduce the impact of uncertainty and individual variations. Further analysis was conducted on some FEGs and PSGs of interest. 

**Figure 3:**
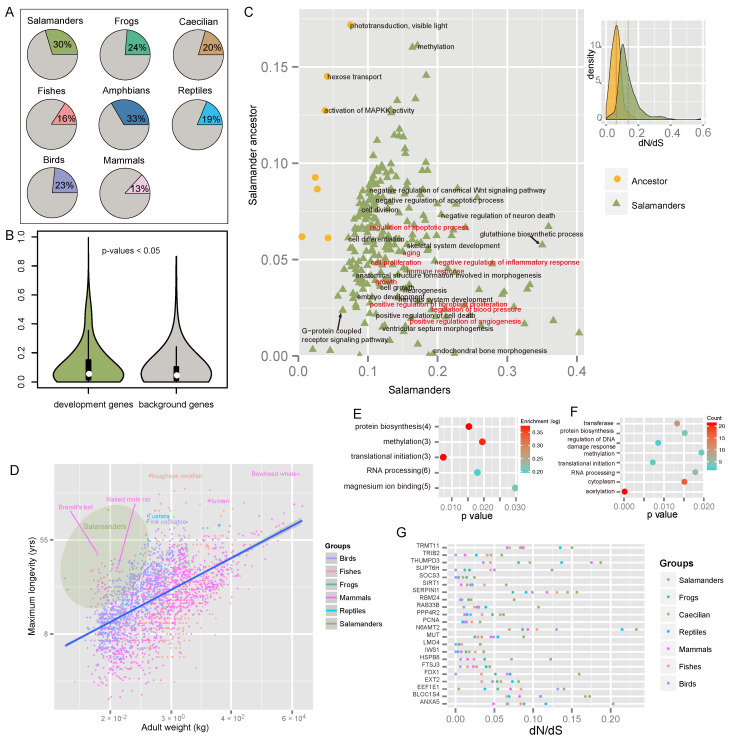
Genetic potential underlying salamanders’ regenerative capability and longevity. (**A**) Proportion of rapidly evolving GO categories (REGOs) involved in development is highest in salamanders. (**B**) Salamanders’ developmental genes evolved significantly faster than other genes. (**C**) Extant salamanders’ development-related and aging-related GO categories evolved significantly faster than those of their ancestors. (**D**) Salamanders fall into the category of exception to the rule of “Larger animals live longer”, which is shown by the yellow-green color. (**E**) Top five enriched GO terms from salamanders’ fast-evolving genes (FEGs). (**F**) Top eight GO terms for salamanders’ FEGs. (**G**) Selected salamanders’ FEGs may be responsible for regenerative capability and longevity.

To investigate the lineage-specific evolution of function modules in the genome, I identified rapidly evolving GO categories (REGOs) unique to each major group of vertebrates. For Lissamphibia, many REGOs were associated with developmental processes, including the development of the circulatory (blood circulation, regulation of blood pressure), respiratory, sensory, and immune systems and morphogenesis (Figure 2E). Developmental process-related REGOs accounted for a higher proportion in Lissamphibia (33%) than in other vertebrate groups (13–19%), including birds who have highly specialized anatomic structures (23%) (Figure 3A).

### 3.5. Regeneration- and Development-Related Genes in Salamanders

Salamanders have a superior ability to regenerate limbs compared to other vertebrates. I specifically examined genes and GO categories that are linked to development and regeneration in salamanders. I also compared salamanders to another vertebrate, the green anole, which also has a regeneration ability.

**Figure 4:**
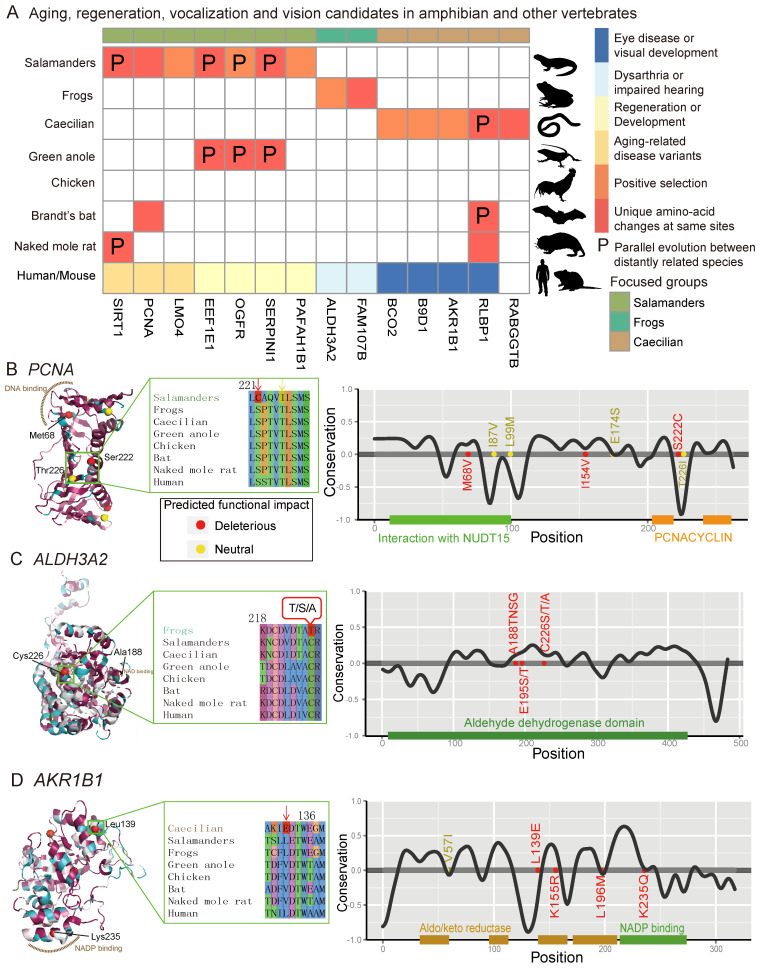
Aging, regeneration, vocalization, and vision candidates in extant amphibians and other vertebrates. (**A**) Candidates under positive selection were identified, and those with shared common or unique position mutations in focused lineages and who have undergone parallel evolution are marked. Associated diseases are also labeled with different colors. (**B**–**D**) Salamander, frog, and caecilian candidates, respectively. **Left**: Crystal structure of candidates. Purple-red represents conserved regions, and cyan represents variable regions. The important domains and lineage-specific mutations are highlighted in the structure. Residue color represents the strength of the functional impact. Insert: alignment of an example residue with functional impact. **Right**: The conservation scores, domain, and lineage-specific mutation positions. The black curve shows the cubic smoothing spline of the amino acid conservation scores; higher scores indicate higher conservation.

The regulation of apoptosis activity plays a key role in local cell dedifferentiation [108,109], which is the first and perhaps the most crucial stage of regeneration. Not surprisingly, I detected several rapidly evolving GO categories associated with the regulation of apoptotic pathways that are unique to salamanders (Figure 2E). Notably, one REGO of regulation of apoptotic cell death was triggered by the tumor suppressor p53 (Figure 2E), which has been proven to be critical for regeneration [110]. In addition, a fast-evolving gene, *EEF1E1*, which is involved in the negative regulation of cell population proliferation and positive regulation of apoptotic processes, shows signals of parallel evolution between salamanders and green anoles (Figure 4A; L108F; *p* < 1 × 10^−6^). Furthermore, some REGOs were involved in macrophage regulation and the MHC biosynthetic process (Figure 2E). This is consistent with the essential role of macrophages in successful healing and regeneration [8]. Many FEGs in salamanders, such as *ANXA5*, *SIRT1*, *RAB33B*, *HSPB8*, etc. (Figure 3G), may be involved in the coagulation process, inflammatory processes, and autophagy during the initial stage of regeneration.

Unlike mammals, salamanders have a scar-free healing capacity, mainly due to their fibroblasts, which form the early blastema rather than scars and control the regeneration process [111]. I detected positive selection acting on the salamanders’ *PAFAH1B1* gene, which plays a crucial role in the directed migration of fibroblasts during wound healing and stem cell division [112]. Furthermore, two amino acid changes (L37F and I200V) were specific to salamanders. L37F is located in the LisH domain, which may be required to activate dynein, and I200V is in the WD40 repeat; both mutations were predicted to have strong functional impacts. In addition, the ERK pathway, whose activity is another key difference in cellular reprogramming between salamanders and mammals [9], and the retinoid metabolic process, which may play an important role in the early phase of regeneration [113], were also found to have undergone the most rapid evolution in salamanders (Figure 2E).

The second stage of regeneration is similar to the development process in that it involves proliferation, redifferentiation, and growth. I detected several development-related REGOs that were unique to salamanders, including anatomical structure formation, neuromuscular process, hedgehog receptor activity, and vasculature development, among a few others. (Figure 2E). I detected a strong signal of natural selection on a cell proliferation-related gene, LMO4. One site in its LIM-type zinc finger (Znf) domain (region 23–83), which acts as an interface for protein–protein interactions, was under positive selection (Ala24; Bayes empirical Bayes posterior probabilities (BEBPP) = 0.996). I also detected seven FEGs involved in differentiation and development, including *TRIB2*, *RBM24*, *EXT2*, *SOCS3*, *BLOC1S4*, *MUT*, and *SERPINI1* (Figure 3G and Table S1). Furthermore, the gene *SERPINI1*, which is associated with central nervous system development, was detected to have undergone parallel evolution between salamanders and green anoles (Figure 4A; *p* < 1 × 10^−6^). A parallel change (F118Y) was predicted to be harmful. The prostaglandin biosynthetic process, a pathway that was recently reported to be closely associated with regenerative capacity in mice (*15*-*PGDH*) [114] and lizards (*PTGIS* and *PTGS1*) [115], was detected to have evolved most rapidly in salamanders (Figure 2E; *PTGES2* and *PTGS2*). The mutation position of *PTGES2* was shared among salamanders (E183A), echolocating bats (E183D), and naked mole rats (E183D). Unlike *PTGS1*, which is constitutively expressed, *PTGS2* is upregulated during inflammation and contributes to cell proliferation, angiogenesis, apoptosis inhibition, and immune response suppression [116].

Furthermore, I found that the REGOs related to development in salamanders were the most common group in vertebrates (Figure 3A). Did the development-related genes drive the accelerated evolution of salamanders and contribute to regeneration? The answer seems to be yes, as the evolutionary rate of development- and regeneration-related genes were significantly higher than that of the other background genes (Figure 3B; *p* = 0.039). In contrast, a similar pattern was not observed in any other vertebrate group (Figure S9).

How did salamanders obtain such an amazing regenerative ability? To answer this question, I compared the extant salamanders with their most recent common ancestors (MRCAs). Interestingly, many functional modules pertaining to the regeneration process, including the apoptotic process, immune responses, proliferation, angiogenesis, growth, morphogenesis, and aging, have undergone faster adaptive evolution in the extant salamanders relative to their MRCAs (Figure 3C). This is to say, salamanders were constantly evolving their regenerative capacity. Continuous self-improvement may have helped salamanders improve their genetic potential to heal wounds after injury and also delay aging. For example, the opioid growth factor receptor (*OGFR*) is an important regulator of cell proliferation, tissue growth, cancer, cellular renewal, wound repair, and angiogenesis [117]. In salamanders, *OGFR* likely evolved under positive selection (Figure 4A), and Lys198 was identified as a positively selected site (BEBPP = 0.995). It is intriguing that Val190 and Pro231 of *OGFR* are parallel and common changes in salamanders (V190L, P231K) and lizards that can regenerate their tails (V190L, P231R) (Figure 4A), and all mutations were predicted to probably have a damaging effect on protein function. *OGFR* can interact with *TERF2IP*, a regulator of telomere length that is tightly bound to aging, raising a potential correlation between regeneration ability and aging.

**Figure 5:**
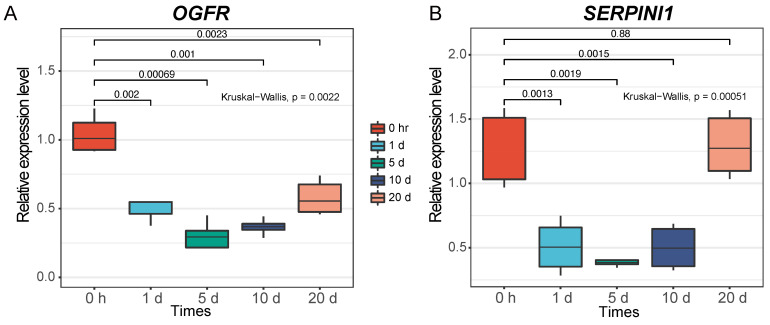
Expression patterns measured by qPCR during the time course of limb regeneration. (**A**) *OGFR*. (**B**) *SERPINI1*. Two biological replicates, each with three technical replicates, were measured at each time point.

To investigate the potential effect of *OGFR* and *SERPINI1* on regeneration, I used real-time quantitative PCR (qPCR) to measure their expression levels in the healing limb blastemata of Chinese fire-bellied newts (*Cynops orientalis*) at 0 h, 1 day, 5 days, 10 days, and 20 days post-amputation. The expression of both genes varied significantly at almost every time point compared to that at 0 h (Figure 5). Both genes exhibited similar trends along the time course, with gradually decreasing expression over time, reaching a minimum around the fifth day, and then gradually increasing expression. The expression level of one gene, *SERPINI1*, on the 20th day was almost restored to that at 0 h. Previous studies found that activation of *OGFR* prevents cell proliferation [118], and knockdown of *SERPINI1* reduces the outgrowth of neurons [119]. Thus, the observed fluctuations in expression levels over time may point to a dynamic regulation of cell proliferation and axonal growth during limb regeneration.

This study identified several critical genes that are linked to regeneration. Many of these genes were expected and/or consistent with the findings of previous studies [111,120]. Interestingly, three genes (*EEF1E1*, *OGFR*, and *SERPINI1*) displayed significant signals of parallel evolution in salamanders and lizards, both of which possess regenerative capacities, implying a functional convergence for their regenerative mechanisms. Specific changes in these candidates in salamanders may explain, in part, why humans cannot regrow perfectly like salamanders. It is well known that some genes are only activated in specific tissues and/or conditions, such as in healing tissues [121]. Therefore, some genes may have been missed in my screening, which is a limitation of this study. Although I validated two regeneration-related candidates (*OGFR* and *SERPINI1*) by qPCR in healing blastemata and confirmed that their expression levels change significantly during limb regeneration, this was a limited solution.

### 3.6. Longevity-Related Genes in Salamanders

Lifespan commonly correlates with body mass for most animals. This rule is named “Larger animals live longer”, although there are several exceptions in mammals, fish, reptiles, and birds (Figure 3D). It is noteworthy that this is not the case for most salamanders (Figure 3D). Olm (*Proteus anguinus*) is possibly the longest-living salamander, which can live over 100 years, even though its body mass is only about 20 g. In contrast, the longest-living mammal (bowhead whale) can live over 200 years but with a 100,000,000 g weight. The average lifespan of salamanders is about 18 years, calculated based on 70 species of salamanders, which is significantly higher than that of frogs (about 12 years; WRST *p* = 2.9 × 10^−5^) in the case where there is no significant difference in body mass between the sister groups (WRST *p* = 0.219).

How do salamanders extend their life span? The “rate of living” (ROL) theory of aging (the faster the metabolism, the shorter the lifespan) may partly answer this question (Figure S10; Spearman correlation = −0.65, *p* = 7.5 × 10^−45^), although there are numerous outliers. Salamanders possess the lowest metabolic rates among tetrapods [122], which can greatly help them reduce the accumulation of damage from reactive oxygen species (ROS) in the body and slow aging. Furthermore, the lowest metabolic rates may also lead to the extremely low mutation rates in salamanders due to the link between the frequency of oxidative damage and the likelihood of DNA change [123]. In other words, longevity seems to be negatively correlated with the mutation rate, which is similar to longer-lived rockfish (although the authors mainly focused on mitochondrial mutations) [124].

It is now clear that the genetic mechanisms underlying aging can be conserved across distantly related species [125]. Increasing evidence indicates that epigenetic factors have critical roles in aging [126]. I examined genes and GO categories that are related to epigenetics and compared them between salamanders and other long-living vertebrates.

A large number of REGOs in salamanders are involved in histone modification, particularly methylation and acetylation, and were significantly enriched (Figure 3E,F). The FEGs included *SIRT1*, *IWS1*, *SUPT6H*, *FTSJ3*, *THUMPD3*, and *TRMT11* (Figure 3G).

I further examined several candidate genes in more detail. *SIRT1*, also known as NAD-dependent deacetylase sirtuin-1, plays a critical role in metabolic regulation. It is an important genetic modulator for aging and longevity in humans, mice, worms, flies, and yeast [127]. *SIRT1* evolved much more rapidly in salamanders (dN/dS = 0.065) than in any other animals (dN/dS ≤ 0. 037). I uncovered two interesting amino acid changes occurring at crucial regions of *SIRT1* in salamanders. One mutation (K375R) located in the catalytic core small domain (365–417) of the sirtuin family domain (deacetylase sirtuin-type), a key component responsible for NAD+ binding and the histone deacetylation reaction (associated with aging), was predicted to be possibly harmful to protein function. In addition, K375R is a parallel change that occurred in both salamanders and the long-lived and cancer-resistant naked mole rat. It is possible that K375R may be directly related to longevity in salamanders. Another salamander-specific mutation (I227L) is in a nuclear localization signal (223–230) and a region that is the site of interaction with *CCAR2* (*DBC1*), a partner that can inhibit *SIRT1* deacetylase activity. I227L may decrease the interaction between *SIRT1* and *CCAR2* and delay aging. In addition, *SIRT1* is involved in the insulin-like signaling (ILS) pathway, which evolved rapidly in salamanders and is a metabolic signaling pathway involved in controlling life span in many species [125]. *SOCS3*, another FEG, is also involved in ILS regulation (Figure 3G). Notably, *IWS1* and *SUPT6H*, both evolving the fastest in salamanders, interact with each other and form a complex to control histone modifications [128]. In addition, *IWS1* has at least eight salamander-specific mutations (K581R, E605D, V612A, S646G, K653R, T700S, R716K, and V763L).

**Figure 6:**
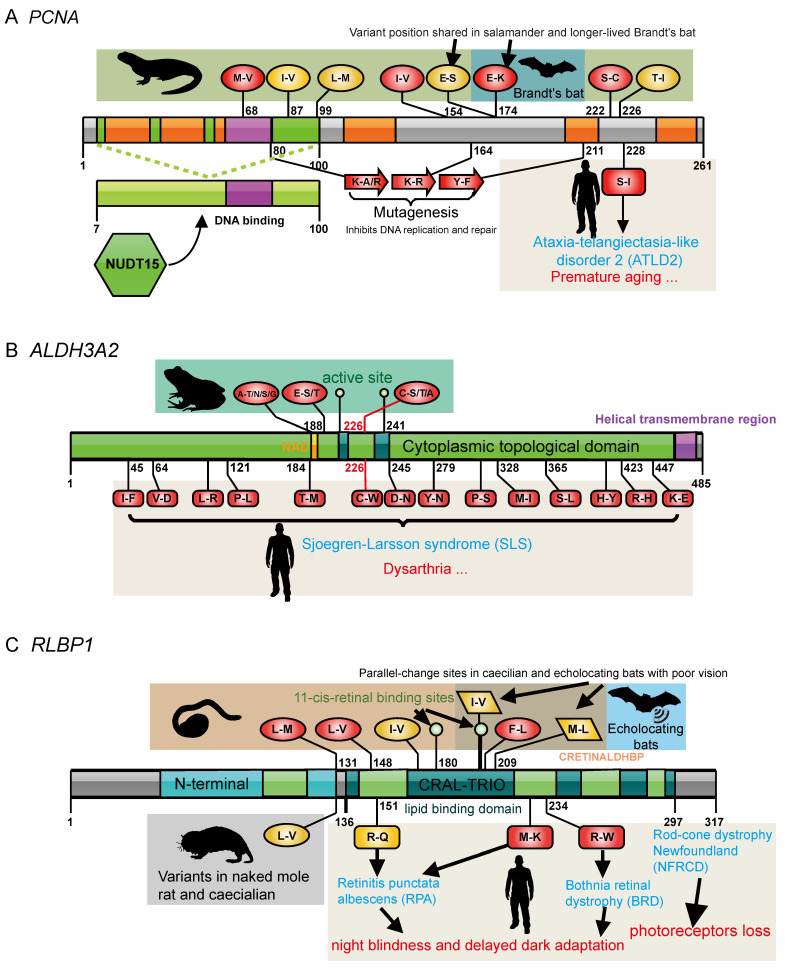
Specific and shared variants of candidate genes in extant amphibians and distantly related vertebrates with similar traits. (**A**) PCNA in salamanders. (**B**) ALDH3A2 in frogs. (**C**) RLBP1 in caecilians. Color of residue represents the strength of the functional impact. Red: harmful; yellow: neutral.

The DNA damage response and repair, which counter the constant assaults by endogenous and environmental agents on DNA, are critical for maintaining genetic stability and are considered to be the key to aging and longevity. I detected that DNA repair-related GOs evolved most rapidly in salamanders (Figure 2E; *PCNA*, *SIRT1*, and *PPP4R2*). Proliferating cell nuclear antigen (*PCNA*), which is essential for DNA replication and damage repair [129], was associated with aging in humans, rats, and long-lived bowhead whales [130,131]. It is highly conserved even between plants and animals, indicating a strong selection pressure for structure conservation in order to interact with its partners. Correspondingly, numerous mutations (Figure 6A) in *PCNA* resulted in impaired DNA replication and damage repair [132]. Notably, salamander *PCNA* evolved at a rate more than two times faster than any other vertebrates (Figure 3G; and Table S1), which results in up to seven unique amino acid mutations present in all salamander species, and of these, three were predicted to be deleterious (Figure 4B and Figure 6A).

It is worth noting that three amino acid substitutions (M68V, I87V, and L99M) are located in the region that interacts with *NUDT15* (Figure 6A), a partner that is important in protecting *PCNA* from degradation [133]. One important mutation (M68V) is in the conserved DNA-binding region (61–79) and was considered to be deleterious (Figure 4B and Figure 6A). These three changes were expected to strengthen the physiological interaction between *PCNA* and *NUDT15* and enhance the stability of *PCNA* in salamanders. It has been shown that the S228I mutation in humans significantly decreases *PCNA* interactions with *FEN1*, *LIG1*, and *ERCC5* and can give rise to an age-related syndrome (ataxia telangiectasia-like disorder 2 (ATLD2)) in which the clinical features include premature aging, a short stature, development delay, neurodegeneration, and hearing loss due to impaired DNA repair (Figure 6A) [134]. Here, I uncovered two salamander-specific amino acid mutations (S222C and T226I) near position 228, and S222C was predicted to have a damaging effect on *PCNA* structure (Figure 4B). Another interesting position in *PCNA* is 174, which is a well-conserved position in vertebrates because common changes were found in salamanders (E174S) and the longer-lived Brandt’s bat (E174K) (Figure 6A). In contrast, this is not the case for other vertebrates, even for normal-lived bats (including the little brown bat, vampire bat, David’s myotis, and black flying fox). Considering the key role of *PCNA* in the DNA damage response, these amino acid changes may be directly related to minimizing the negative effects of ROS, which aid in slowing the aging of salamanders. Moreover, it is well known that telomeres shorten with age, which is in part because of the end replication problem. *PCNA* may contribute to the maintenance of proper telomere length via semi-conservative replication. Moreover, although salamanders’ aging-related module evolved significantly faster than that of their MRCAs (Figure 3C), the DNA repair process did not, raising the possibility that salamanders’ MRCAs already possessed a stronger ability to repair DNA damage.

It is intriguing that the sensory perception of pain evolved much faster (greater than two times) in salamanders than in any other vertebrate (Figure 2E). A recent study indicated that loss of pain perception can directly delay aging via knocking out *TRPV1* pain receptors [135]. I studied *PTGS2*, a regenerative candidate that was mentioned previously, which is also responsible for the sensory perception of pain. This study’s findings once again suggest that longevity and regeneration are likely to be closely related. Another similar case is *LMO4*. *PCNA*, *EEF1E1*, *OGFR*, and *SIRT1* also play dual roles in life expectancy and regenerative capacity. With this in mind, it is not difficult to imagine how salamanders can perfectly regenerate complex structures, even when aging.

### 3.7. Vocalization- and Hearing-Related Genes in Frogs

Vocalization is the primary form of communication of most frogs during their breeding season. I detected several positively selected genes that are potentially linked to vocalization and hearing.

The gene fatty aldehyde dehydrogenase (*ALDH3A2*) bore the signatures of positive selection. *ALDH3A2* is a critical gene associated with Sjögren–Larsson syndrome (SLS), which is characterized by dysarthria and a few other symptoms in humans. The three sites of *ALDH3A2* (Ala188, Glu195, and Cys226) that were under natural selection in frogs awakened my interest. These sites are located in the highly conserved aldehyde dehydrogenase domain (Figure 4C), and Ala188 is in a small core region (185–190) of the NAD-binding domain (Figure 6B). Importantly, amino acid changes at position 226, which varied in frogs, can cause SLS in humans (Figure 6B). Furthermore, many other variants in *ALDH3A2* can also lead to SLS [136], implying the importance of conserved sites to maintain the function of *ALDH3A2*.

*FAM107B* is a candidate associated with the sensory perception of sound. For frog *FAM107B*, I detected a unique amino acid change (A46S), which probably has a harmful effect on protein structure. I also observed three unique amino acid mutations in *FAM107B* in naked mole rats (H34R, L37F, and Q41L), who have lost much of their ability to localize sounds due to their subterranean lifestyle. In addition, *FAM107B* knockout mice exhibit impaired hearing [137]. Furthermore, TIMM10, which is involved in the sensory perception of sound, evolved the fastest in frogs (Table S1).

### 3.8. Vision-Related Genes in Caecilians

Similar to naked mole rats, most caecilians live a subterranean lifestyle and have degenerated visual functions. I examined the molecular basis for the poor vision of caecilians and identified the top nine candidates. These included six genes that are potentially involved in visual perception/stimulus and/or the retinoic acid metabolic process, including three PSGs (*BCO2*, *JUNB*, and *CALR*), two FEGs (*RABGGTB* and *CLN5*), and one parallel-evolved gene (*RLBP1*).

The retinaldehyde-binding protein 1 (*RLBP1*) gene revealed an interesting pattern of parallel evolution. *RLBP1* is a crucial visual protein expressed in the retinal pigment epithelium and is involved in the retinal “visual cycle” [138]. Mutations in *RLBP1* cause rod–cone dysfunction and severe vision loss, and they are associated with numerous eye diseases such as night blindness, delayed dark adaptation, and loss of color vision (Figure 6C). I observed six unique missense mutations in *RLBP1*, of which five are located in the CRAL-TRIO domain, a hydrophobic binding pocket for 11-cis-retinal binding (Figure 6C). All known mutations in the CRAL-TRIO domain have been proven to cause a series of severe visual diseases due to impaired 11-cis-retinal binding and release triggered by *RLBP1* structural transitions [139]. Furthermore, I detected one common mutation in *RLBP1* between caecilians (L131M) and naked mole rats (L131V; Figure 6C) and two parallel changes between caecilians and echolocating bats (Brandt’s bat, little brown bat, and David’s myotis; I201V and M209L, *p* = 0.000894), which all live in dim-light environments and display a reduced visual capacity. Interestingly, a non-echolocating bat, the black flying fox, which has excellent eyesight, did not have changes at Ile201 and Met209. Considering the essential role of *RLBP1* in the conversion of photobleached opsin molecules into photosensitive visual pigments, these shared changes may be responsible for the complete or partial color blindness of these species and imply a convergent evolution due to their dark habitats.

A PSG, *AKR1B1*, was detected with the positively selected sites Val57 (BEBPP = 0.975) and Leu139 (BEBPP = 0.982). It is an important gene associated with retinal disease and cataracts in humans. It is noteworthy that all the caecilian-specific deleterious mutations occurred in important regions, especially the NADP-binding motif, which is located in a large, deep, elliptical pocket in the C-terminal end of *AKR1B1* (Figure 4D). A parallel site mutation (T136A, *p* = 0.548152) was only detected in caecilians and echolocating bats but not in non-echolocating bats. Another two PSGs (*B9D1* and *KLF4*) participate in camera-type eye development, whereas positive selection sites (Gln21 for *B9D1*, BEBPP = 0.998; Ser421 for *KLF4*, BEBPP = 0.994) and unique deleterious amino acid changes (T73S for *B9D1* and S421N for *KLF4*) may be associated with the small-sized eyes of caecilians.

## 4. Conclusions

To conclude, this study provides new insights into the origin of Lissamphibia and the genetic basis of adaptive traits of extant amphibians, particularly the regeneration ability and longevity of salamanders. The discovery of these critical genes will set the stage for further functional analyses. With the recent developments in gene editing technology, the importance and function of these candidate genes can be tested, which will provide much-needed clues to understanding the processes of regeneration and aging. All of these will open new avenues to understanding their genetic systems and to exploiting the genetic potential of humans and improving human well-being.

## Data Availability

Transcriptome sequencing data were deposited in the Genome Sequence Archive (GSA) of the National Genomics Data Center (NGDC) at https://ngdc.cncb.ac.cn/gsa/ (accessed on 10 August 2023) under accession number PRJCA018958.

## Supplementary Materials

The following supporting information can be downloaded at: https://www.mdpi.com/article/10.3390/ani13223449/s1, Figure S1. Phylogenetic hypothesis about origin and relationship of three orders of extant amphibians. (A) Monophyletic origin: Batrachia hypothesis (H1); Procera hypothesis (H2); frog–caecilian sister relationship (H3). (B) Paraphyletic origin: caecilian–amniote and salamander–frog relationships (H4); (salamanders (caecilians, amniotes)) (H5); Figure S2. Completeness score and information content. (A) Completeness score of species pairwise comparison. A high score represents a high degree of shared site coverage (green), while a low score represents a low degree of shared site coverage (red). (B) Information content of potential phylogenetic signal for each gene. Dark blue represents a high information content, light blue represents a low information content, and red (0) represents no information content; Figure S3. Density plot showing degree of compositional heterogeneity for each gene. (A) Amino acid sequences; (B) CDS sequences. The lower the RCFV value, the lower the degree of compositional heterogeneity in that gene; Figure S4. Global stationary, reversible, and homogeneous (SRH) conditions of species pairwise comparison for different datasets (A-I). The higher the *p*-value, the more consistent its evolution with evolution under SRH conditions; Figure S5. Supporting conditions for the five phylogenetic hypotheses concerning the relationship among the three orders of extant amphibians. (A) AU test based on amino acid sequences. Each vertical line represents a gene, and different colors represent different *p*-values from the AU tests. A small *p*-value (dark green) indicates that a gene rejects a topology. (B) AU test based on CDS sequences. (C) Venn diagram based on amino acid sequences. Numbers in brackets represent the quantity of genes that cannot reject a specific hypothesis. (D) Venn diagram based on CDS sequences; Figure S6. Estimation of origin and divergence date of extant amphibians. (A) Clock of protein. (B) Clock of 2nd codon of CDS. Numbers on nodes are age estimations, and blue bars represent 95% confidence intervals. Time unit is ten billion years. Fossil records are in red point on node; Figure S7. Estimation of mutation rates of the synonymous sites (dS). (A) Boxplot of dS values for each major vertebrate group. Red point is the mean value of the dN/dS ratio. Salamanders’ dS ratios were significantly smaller than any other vertebrates (*p* < 2.9 × 10^−40^ for all pairwise tests). (B) Clustered heatmap of dN/dS ratio of major vertebrate groups. Red color represents a high dN/dS ratio; blue color represents a low dN/dS ratio. (C) dS value tree of 22 vertebrates. Red color represents high dS values, and green represents low dS values; Figure S8. Four-fold degenerate site tree of 22 vertebrates. Color represents mutation rate of 4-fold degenerate sites. Red color represents high rates and green represents low rates; Figure S9. dN/dS ratio comparison of development-related genes and the other genes in three orders of extant amphibians and four other major vertebrates. Wilcoxon rank sum test *p*-values are presented. Only salamanders’ dN/dS ratio of development-related genes was significantly greater than the other genes (*p* = 0.039); Figure S10. Scatter diagram of relationship between metabolic rate and max lifespan; Table S1. Fast-evolving genes for salamanders, frogs, and caecilians; Table S2. Positively selected genes for salamanders, frogs, and caecilians.

## Abbreviations

4D	Four-fold degenerate sites
BEBPP	Bayes empirical Bayes posterior probabilities
BI	Bayesian inference
BIC	Bayesian Information Criterion
CDS	Coding sequence
CH	Compositional heterogeneity
ECM	Extracellular matrix
FDR	False discovery rate
FEGs	Fast-evolving genes
GAC	Genes as characters
GO	Gene Ontology
HaMSTR	Hidden Markov Model-based Search for Orthologs using Reciprocity
LRT	Likelihood ratio test
ML	Maximum likelihood
MPEs	Maximum pseudo-likelihood estimates
PSGs	Positively selected genes
RCFV	Relative Composition Frequency Variability
REGOs	Rapidly evolving GO categories
ROL	Rate of living
SRH	Stationary, reversible, and homogeneous conditions
WRST	Wilcoxon rank sum test
