# Supplementary material for: Evolutionary Insights into the Relationship of Frogs, Salamanders, and Caecilians and Their Adaptive Traits, with an Emphasis on Salamander Regeneration and Longevity
Source: Animals (Basel). 2023 Nov 8;13(22):3449. doi: 10.3390/ani13223449 (PMC10668855; doi:10.3390/ani13223449)
Supplement: Supplementary file 1 [file animals-13-03449-s001.zip › Fig_s2_alistato.pdf]

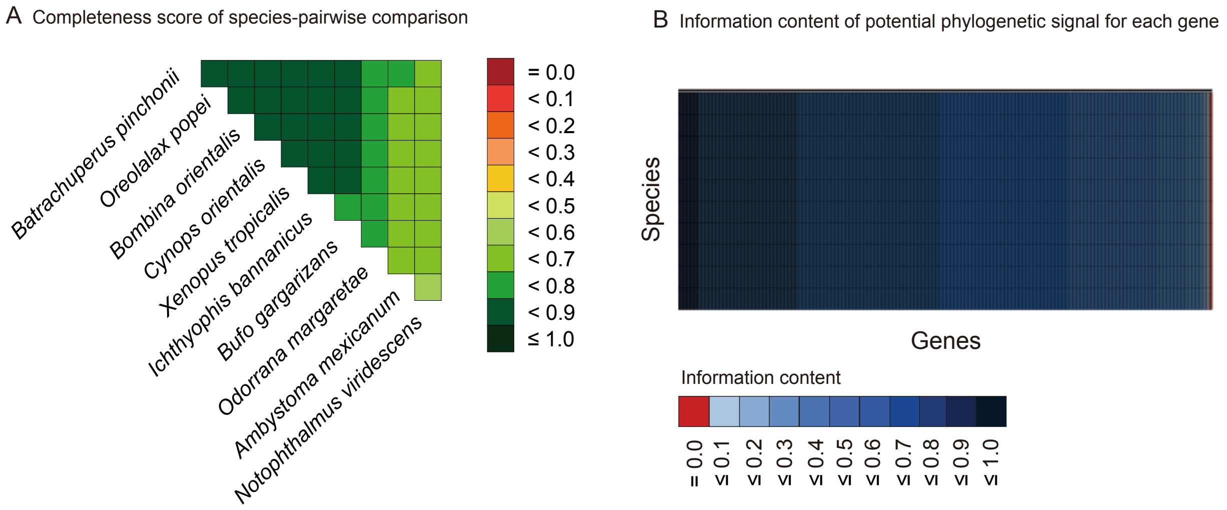
A Completeness score of species-pairwise comparison
Batrachuperus pinchonii
Oreolalax popei
Bombina orientalis
Cynops orientalis
Xenopus tropicalis
Ichthyophis bannanicus
Bufo gargarizans
Odorrana margaretae
Ambystoma mexicanum
Notophthalmus viridescens
= 0.0
< 0.1
< 0.2
< 0.3
< 0.4
< 0.5
< 0.6
< 0.7
< 0.8
< 0.9
≤ 1.0
B Information content of potential phylogenetic signal for each gene
Species
Genes
Information content
= 0.0
≤ 0.1
≤ 0.2
≤ 0.3
≤ 0.4
≤ 0.5
≤ 0.6
≤ 0.7
≤ 0.8
≤ 0.9
≤ 1.0
