# Supplementary material for: Evolutionary Insights into the Relationship of Frogs, Salamanders, and Caecilians and Their Adaptive Traits, with an Emphasis on Salamander Regeneration and Longevity
Source: Animals (Basel). 2023 Nov 8;13(22):3449. doi: 10.3390/ani13223449 (PMC10668855; doi:10.3390/ani13223449)
Supplement: Supplementary file 1 [file animals-13-03449-s001.zip › Fig_s3_Bacoca.pdf]

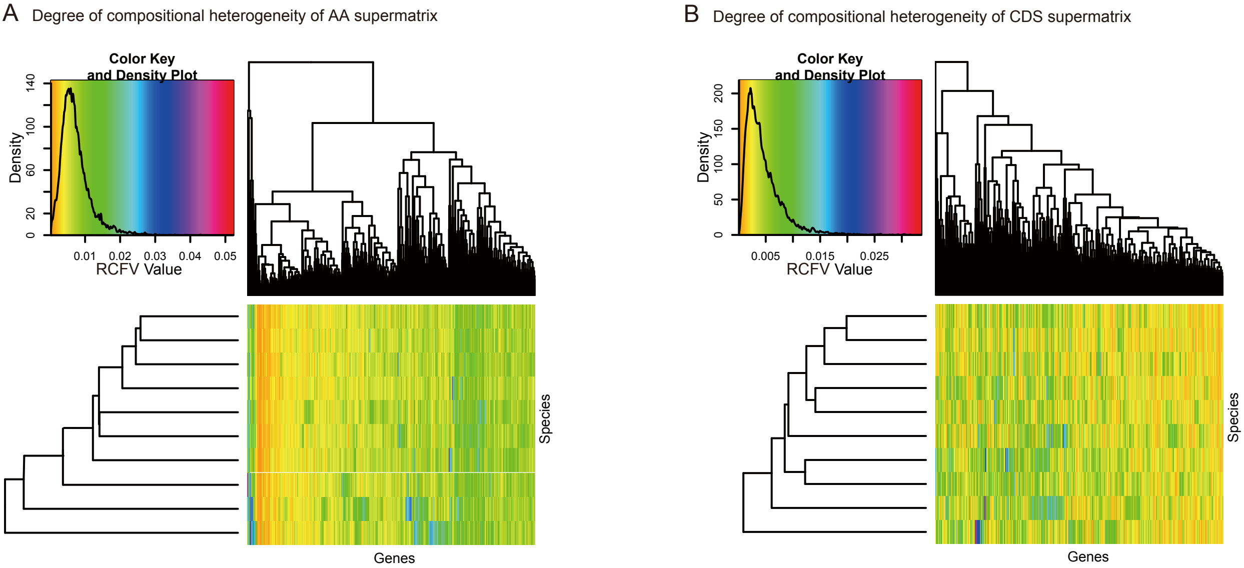
A
Degree of compositional heterogeneity of AA supermatrix
Color Key
and Density Plot
Density
0
20
60
100
140
0.01
0.02
0.03
0.04
0.05
RCFV Value
Species
Genes
B
Degree of compositional heterogeneity of CDS supermatrix
Color Key
and Density Plot
Density
0
50
100
150
200
0.005
0.015
0.025
RCFV Value
Species
Genes
