# Supplementary material for: Evolutionary Insights into the Relationship of Frogs, Salamanders, and Caecilians and Their Adaptive Traits, with an Emphasis on Salamander Regeneration and Longevity
Source: Animals (Basel). 2023 Nov 8;13(22):3449. doi: 10.3390/ani13223449 (PMC10668855; doi:10.3390/ani13223449)
Supplement: Supplementary file 1 [file animals-13-03449-s001.zip › Fig_s4_Symtests.pdf]

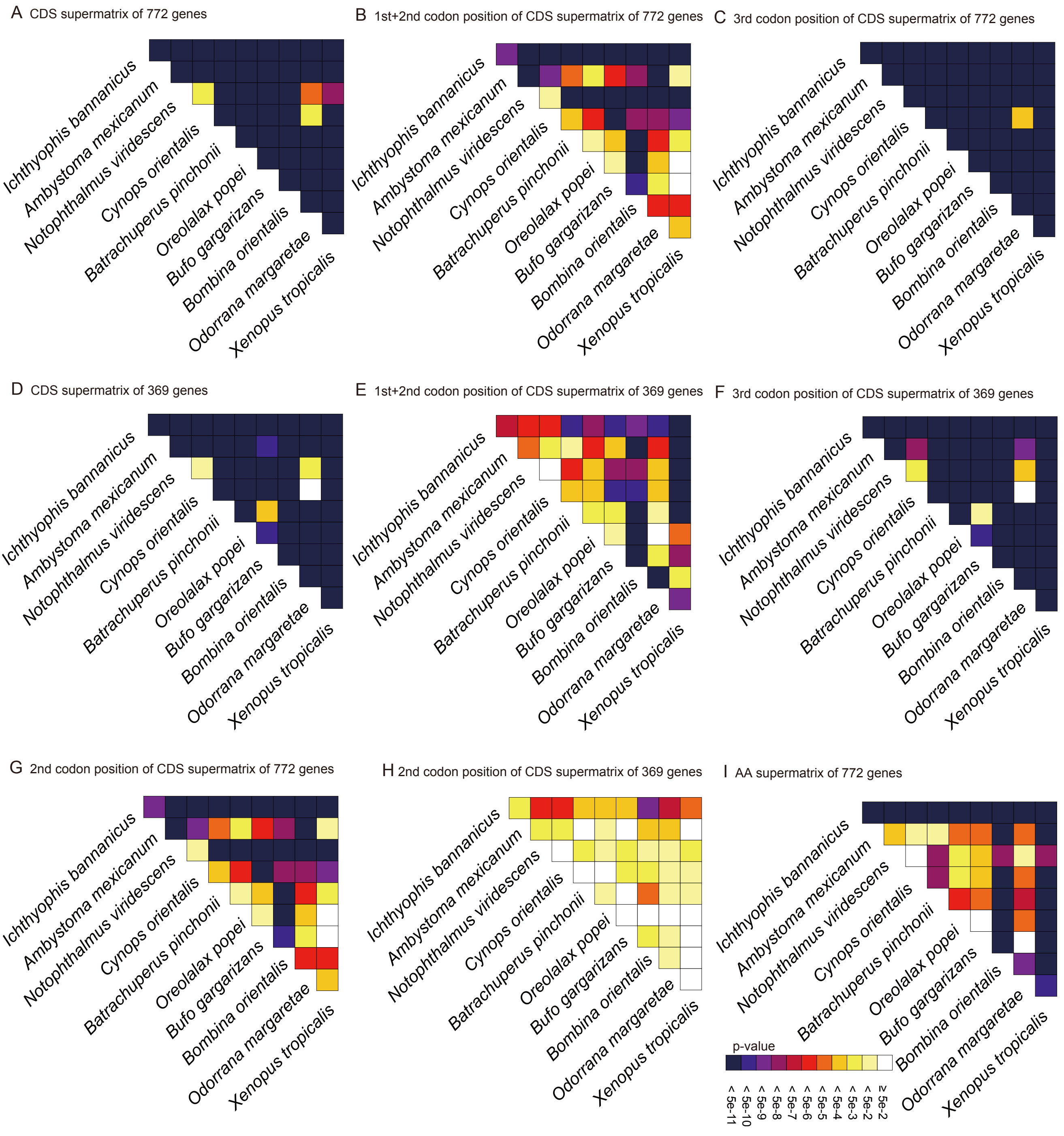
A CDS supermatrix of 772 genes
B 1st+2nd codon position of CDS supermatrix of 772 genes
C 3rd codon position of CDS supermatrix of 772 genes
D CDS supermatrix of 369 genes
E 1st+2nd codon position of CDS supermatrix of 369 genes
F 3rd codon position of CDS supermatrix of 369 genes
G 2nd codon position of CDS supermatrix of 772 genes
H 2nd codon position of CDS supermatrix of 369 genes
I AA supermatrix of 772 genes
Ichthyophis bannanicus
Ambystoma mexicanum
Notophthalmus viridescens
Cynops orientalis
Batrachuperus pinchonii
Oreolalax popei
Bufo gargarizans
Bombina orientalis
Odorrana margaretae
Xenopus tropicalis
p-value
< 5e-11
< 5e-10
< 5e-9
< 5e-8
< 5e-7
< 5e-6
< 5e-5
< 5e-4
< 5e-3
< 5e-2
≥ 5e-2
