# Supplementary material for: Evolutionary Insights into the Relationship of Frogs, Salamanders, and Caecilians and Their Adaptive Traits, with an Emphasis on Salamander Regeneration and Longevity
Source: Animals (Basel). 2023 Nov 8;13(22):3449. doi: 10.3390/ani13223449 (PMC10668855; doi:10.3390/ani13223449)
Supplement: Supplementary file 1 [file animals-13-03449-s001.zip › Fig_s5_CONSEL.pdf]

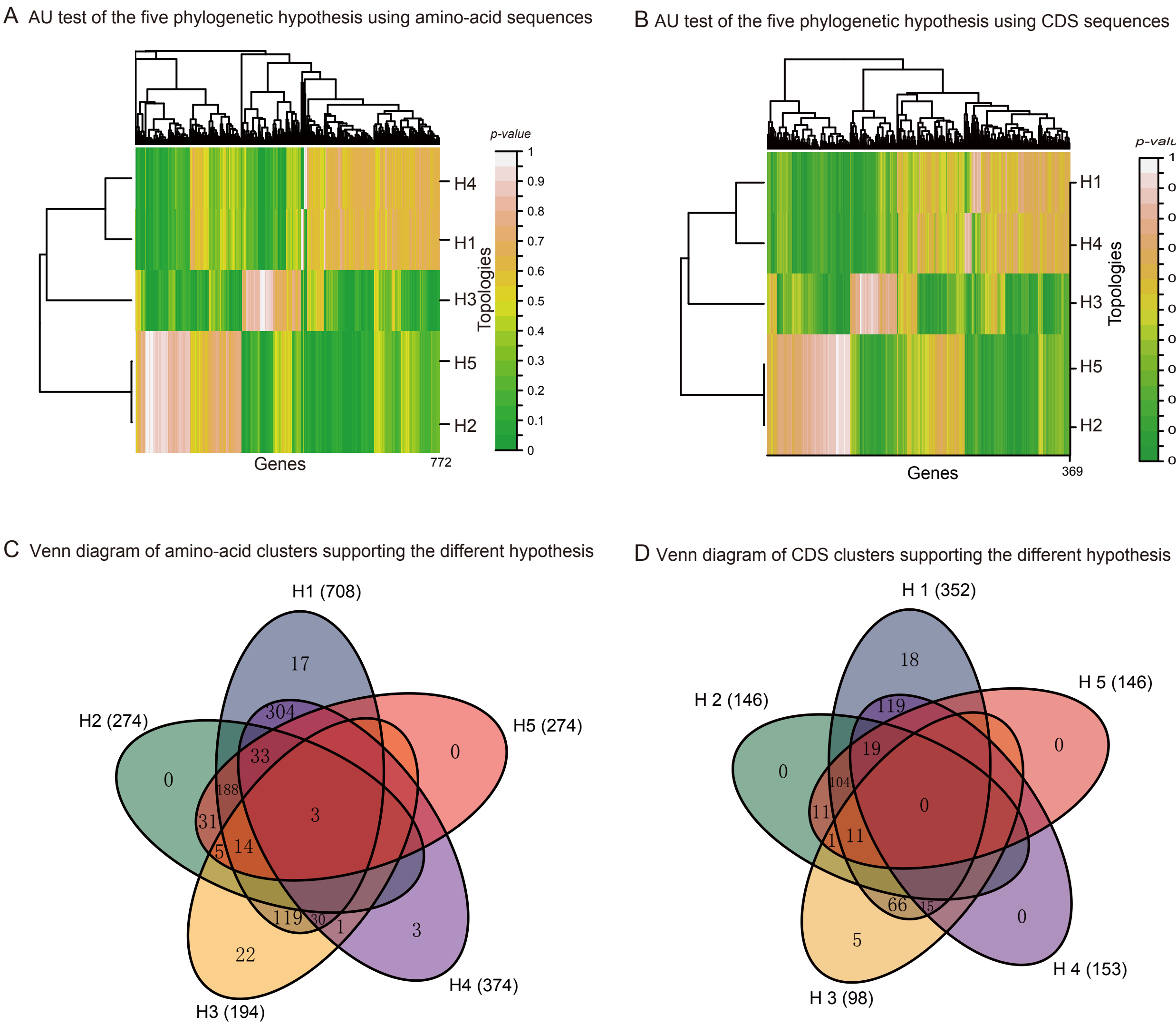
A AU test of the five phylogenetic hypothesis using amino-acid sequences
p-value
1
0.9
0.8
0.7
0.6
0.5
0.4
0.3
0.2
0.1
0
H4
H1
H3
H5
H2
Topologies
Genes
772
B AU test of the five phylogenetic hypothesis using CDS sequences
p-value
1
0.9
0.8
0.7
0.6
0.5
0.4
0.3
0.2
0.1
0
H1
H4
H3
H5
H2
Topologies
Genes
369
C Venn diagram of amino-acid clusters supporting the different hypothesis
H1 (708)
H2 (274)
H5 (274)
H4 (374)
H3 (194)
17
304
0
33
0
188
3
31
5
14
119
30
1
3
22
D Venn diagram of CDS clusters supporting the different hypothesis
H 1 (352)
H 2 (146)
H 5 (146)
H 4 (153)
H 3 (98)
18
119
0
19
0
104
0
11
1
11
66
15
0
5
