# Supplementary material for: Evolutionary Insights into the Relationship of Frogs, Salamanders, and Caecilians and Their Adaptive Traits, with an Emphasis on Salamander Regeneration and Longevity
Source: Animals (Basel). 2023 Nov 8;13(22):3449. doi: 10.3390/ani13223449 (PMC10668855; doi:10.3390/ani13223449)
Supplement: Supplementary file 1 [file animals-13-03449-s001.zip › Fig_s8_phast4d.pdf]

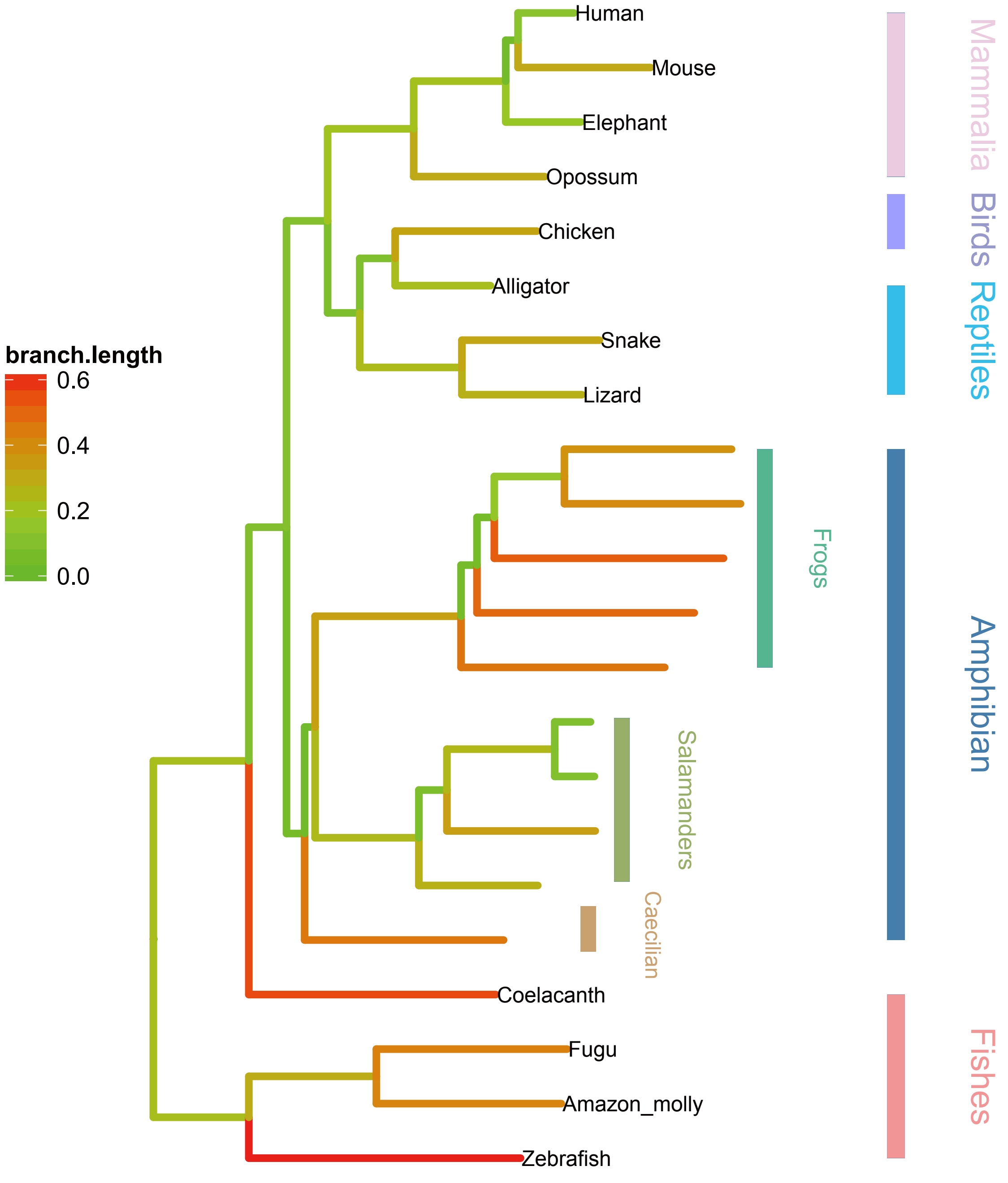
branch.length
0.6
0.4
0.2
0.0
Human
Mouse
Elephant
Opossum
Chicken
Alligator
Snake
Lizard
Coelacanth
Fugu
Amazon_molly
Zebrafish
Frogs
Salamanders
Caecilian
Mammalia
Birds
Reptiles
Amphibian
Fishes
