# Supplementary figures and images for: Evolutionary Insights into the Relationship of Frogs, Salamanders, and Caecilians and Their Adaptive Traits, with an Emphasis on Salamander Regeneration and Longevity
Source: Animals (Basel). 2023 Nov 8;13(22):3449. doi: 10.3390/ani13223449 (PMC10668855; doi:10.3390/ani13223449)

**A** Monophyletic origin

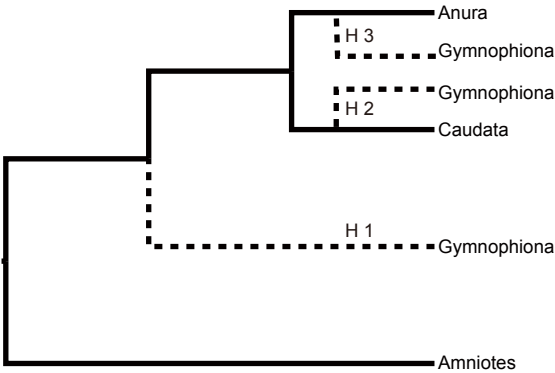

**B** Paraphyletic origin

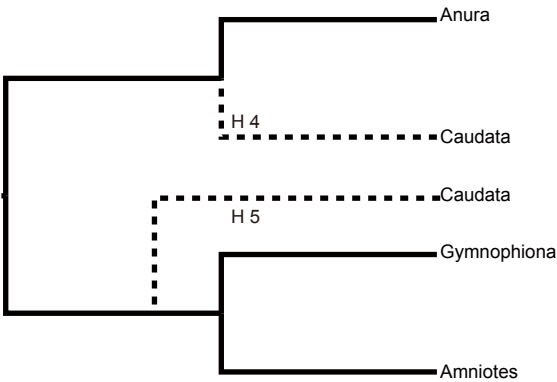

Supplement: Supplementary file 1 [file animals-13-03449-s001.zip › Fig_s1.hypotheses_topologiesAB.pdf]

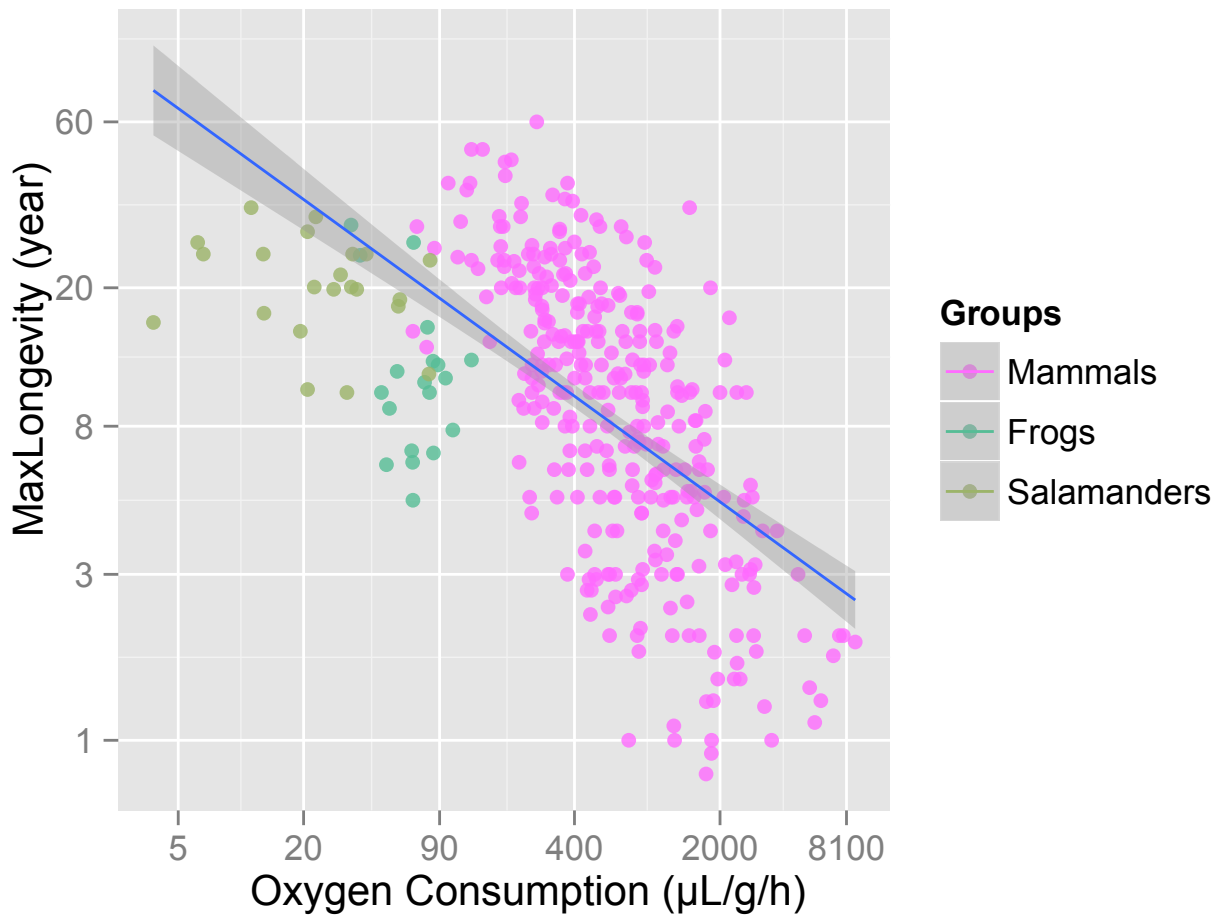

Supplement: Supplementary file 1 [file animals-13-03449-s001.zip › Fig_s10_BMR_LIFESPAN1.pdf]

A pep

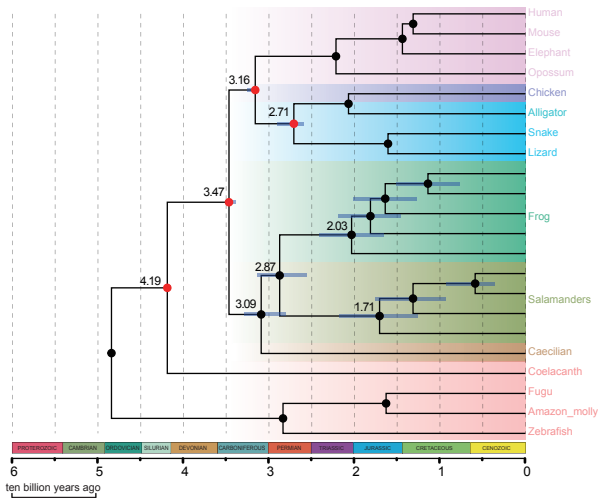

B codon2

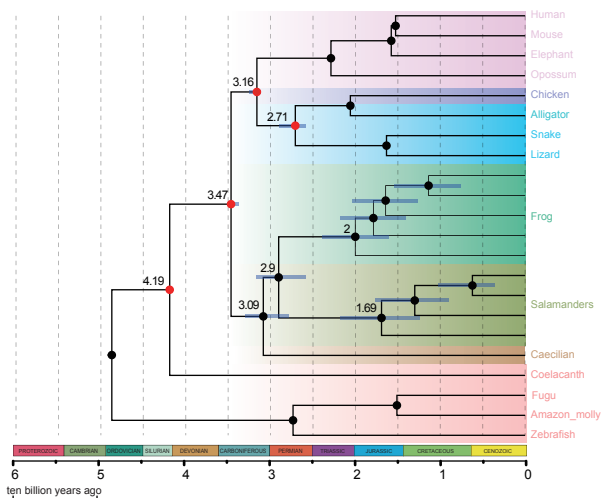

Supplement: Supplementary file 1 [file animals-13-03449-s001.zip › Fig_s6_mcmctree.pdf]

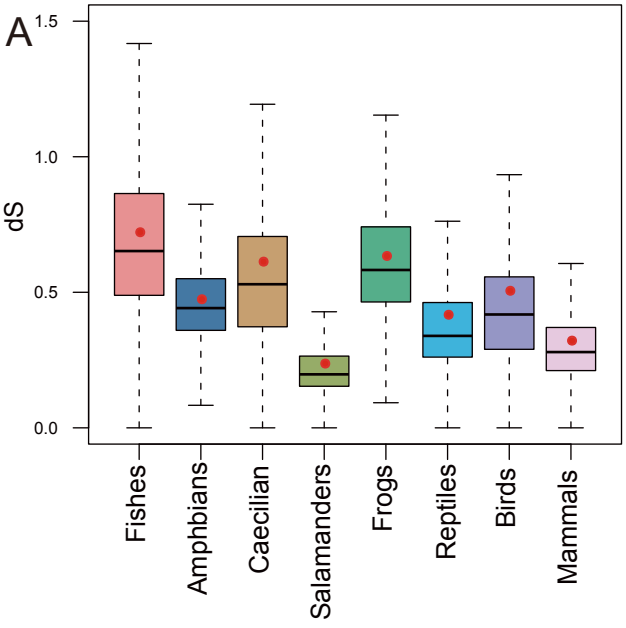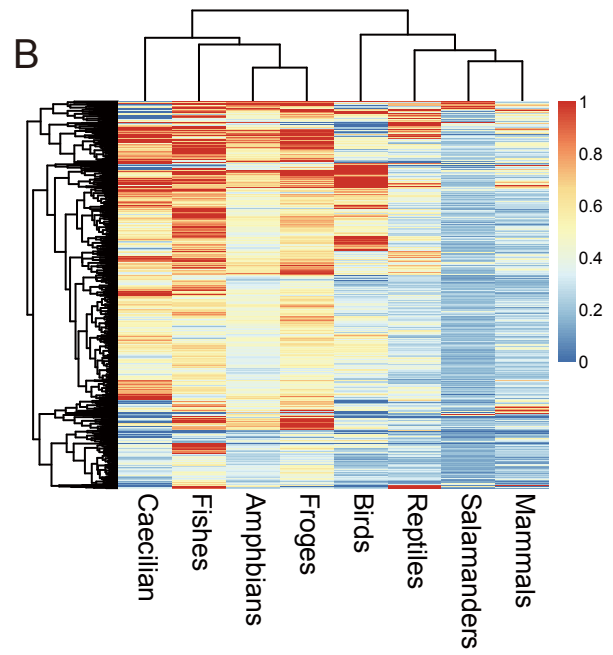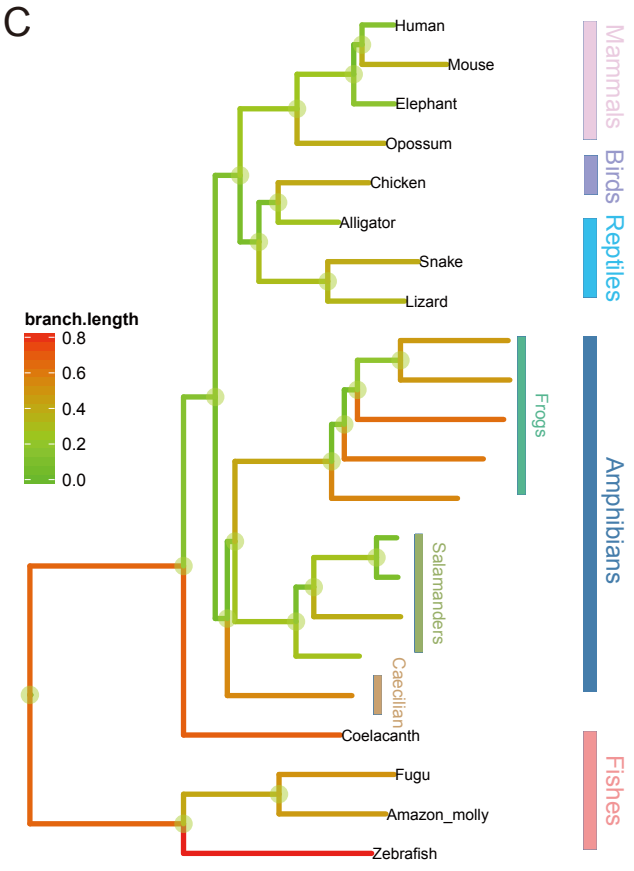

Supplement: Supplementary file 1 [file animals-13-03449-s001.zip › Fig_s7_ds22sp.pdf]

### Salamanders

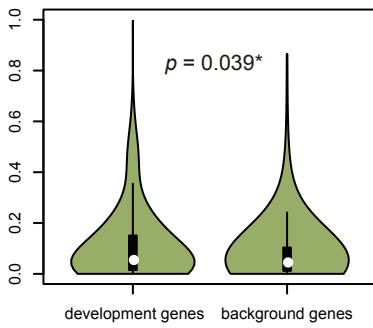

### Frogs

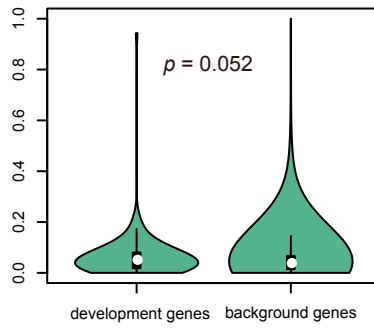

### Caecilian

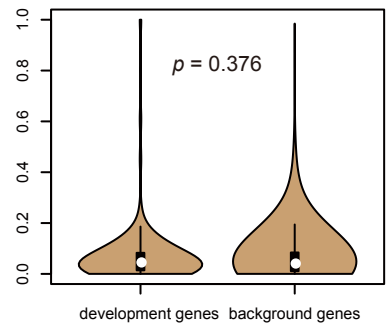

### Fishes

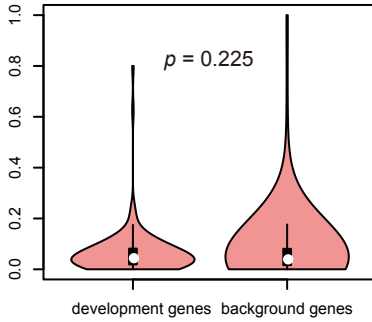

### Reptiles

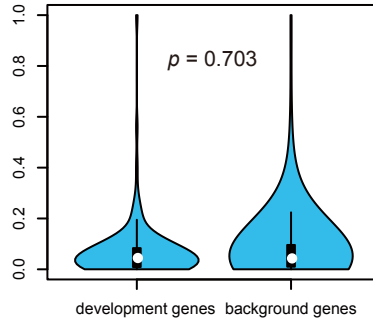

### Bird

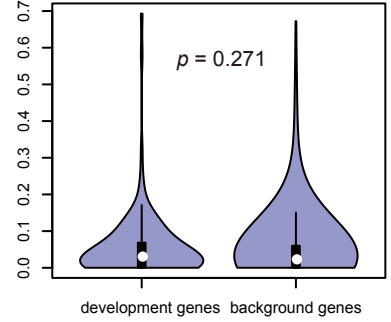

### Mammals

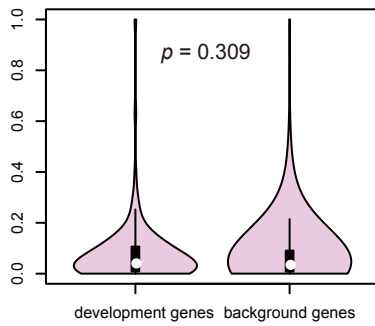

Supplement: Supplementary file 1 [file animals-13-03449-s001.zip › Fig_s9_DEVVSBACK.pdf]
